# Digital Health Technologies for Post-Discharge Care after Heart Failure Hospitalisation to Relieve Symptoms and Improve Clinical Outcomes

**DOI:** 10.3390/jcm12062373

**Published:** 2023-03-19

**Authors:** Paweł Krzesiński

**Affiliations:** Department of Cardiology and Internal Diseases, Military Institute of Medicine—National Research Institute, Szaserow Street 128, 04-141 Warsaw, Poland; pkrzesinski@wim.mil.pl; Tel.: +48-606-939-390

**Keywords:** heart failure, outpatient care, telecare, hospitalisation, digital technologies, e-health, remote monitoring

## Abstract

The prevention of recurrent heart failure (HF) hospitalisations is of particular importance, as each such successive event may increase the risk of death. Effective care planning during the vulnerable phase after discharge is crucial for symptom control and improving patient prognosis. Many clinical trials have focused on telemedicine interventions in HF, with varying effects on the primary endpoints. However, the evidence of the effectiveness of telemedicine solutions in cardiology is growing. The scope of this review is to present complementary telemedicine modalities that can support outpatient care of patients recently hospitalised due to worsening HF. Remote disease management models, such as video (tele) consultations, structured telephone support, and remote monitoring of vital signs, were presented as core components of telecare. Invasive and non-invasive monitoring of volume status was described as an important step forward to prevent congestion—the main cause of clinical decompensation. The idea of virtual wards, combining these facilities with in-person visits, strengthens the opportunity for education and enhancement to promote more intensive self-care. Electronic platforms provide coordination of tasks within multidisciplinary teams and structured data that can be effectively used to develop predictive algorithms based on advanced digital science, such as artificial intelligence. The rapid progress in informatics, telematics, and device technologies provides a wide range of possibilities for further development in this area. However, there are still existing gaps regarding the use of telemedicine solutions in HF patients, and future randomised telemedicine trials and real-life registries are still definitely needed.

## 1. Introduction

The prevention of recurrent heart failure (HF) hospitalisations is of particular importance, as each such successive event may trigger the progression of heart damage, exaggeration of symptoms, reduction in quality of life, and elevation in the risk of death [1,2,3]. Effective care planning and close follow-up during the vulnerable phase after discharge are crucial for symptom control and improving patient prognoses [1,4].

Rapidly developing digital technologies have become increasingly attractive for the outpatient care of patients with HF. Telemedicine solutions, such as video (tele) consultations, structured telephone support, and remote monitoring of vital signs, offer the promise of frequently monitoring HF patients, overcoming potential geographical obstacles, and reducing the burden on healthcare resources [4,5] (Table 1). Electronic platforms provide coordination of tasks within multidisciplinary teams and structured data that can be effectively used to develop predictive algorithms based on advanced digital science, such as artificial intelligence (AI) [6]. Frequent virtual contact with patients provides the opportunity for education and enhancement to promote more intensive self-care.

Theoretically, all of these advantages should reduce HF rehospitalisation by improving patient compliance, allowing for the early detection of clinical decompensation and permitting adequate intervention. The hope for positive effects on mortality is also justified. However, the findings of large individual studies, systematic reviews, and meta-analyses are still incongruous and underscore the need for further research in this area.

## 2. Telecare and Remote Disease Management

Many clinical trials of telemedicine for HF have focused on telecare and remote monitoring, with varying effects on the primary endpoints. The implemented telemedicine models involved different modalities, including teleconsultations, automated voice response systems, video monitoring, transmission of information on symptoms and signs, and telemonitoring of different vital signs [7,8,9,10,11,12,13,14].

The Tele-HF and WISH studies implemented remote transmission of changes in body mass and HF symptoms, and they did not demonstrate any benefits from this type of intervention [7,8]. Additionally, other trials based on nurse telecare [9] and multiparameter monitoring [10,11,12,14] were neutral for their primary endpoints. In the BEAT-HF study, the implementation of remote patient monitoring (blood pressure, heart rate, body mass, and symptoms) failed to reduce all-cause hospital readmission when compared with standard care [11]. Remote intervention (daily body weight measurement, daily recording of HF symptoms, and personalised education) was also ineffective for reducing all-cause mortality and unplanned HF hospitalisations in the OSICAT trial [13].

Some secondary analyses have provided evidence that patients’ compliance with telemedicine interventions strongly influences their outcomes. The post hoc analysis of the BEAT-HF trial [14] proved that each additional day of adherence to weight telemonitoring was associated with a 19% decrease in the rate of death in the following week and an 11% decrease in the rate of hospitalisation. Moreover, participants in the OSICAT trial who were ≥70% adherent to body weight measurement significantly benefited from the telemonitoring programme, as shown by the reduced rate of hospitalisations (hazard ratio (HR) = 0.63, *p* = 0.006) [13].

The TIM-HF trial, which tested remote patient management (RPM) based on complex in-home telemonitoring of vital signs, failed to show a positive effect on mortality (all-cause and cardiovascular) and HF hospitalisations [12]. Conversely, the follow-up TIM-HF2 trial, which was conducted in a population of HF patients with a history of HF hospitalisation in the past 12 months, demonstrated benefits from RPM in reducing the percentage of days lost to unplanned cardiovascular hospitalisations (4.9% vs. 6.6%, HR = 0.80, *p* = 0.046) and all-cause mortality (HR = 0.70, *p* = 0.028) [15]. More recently, Nouryan et al. [16] compared the effects of home telemonitoring and comprehensive outpatient management in 89 outpatients with HF and noted a lower rate of emergency department visits (38% vs. 60%, *p* = 0.040) and better improvement in quality of life in the telemonitoring group (*p* = 0.020), but no significant difference was noted for HF hospitalisations and days of hospital stay. Dawson et al. [17] assessed whether 30-day telemonitoring after discharge, based on home-installed equipment to measure blood pressure, heart rate, pulse oximetry, and weight, would reduce readmissions or mortality in patients at high risk of readmission (*n* = 1380). When compared to the control group, the telemonitoring group was characterised by a lower risk of the primary composite endpoint of hospital readmission or death within 30 days (18.2% vs. 23.7%, *p* = 0.030), as well as a lower rate of emergency department visits (8.6% vs. 14.2%, *p* = 0.005).

In the AMULET trial, an intervention comprising nurse-led non-invasive assessments, telemedicine support, and remote cardiologist decision was applied in outpatients with left ventricular ejection fraction (LVEF) ≤ 49% after an episode of acute HF within the 6 months prior to enrolment. AMULET telecare, when compared to standard care, reduced the risk of the combined primary endpoint of cardiovascular death or first unplanned HF hospitalisation (HR = 0.69, 95% confidence interval [CI]: 0.48–0.99, *p* = 0.044), and this effect was driven mainly by a significant reduction in the risk of a first unplanned HF hospitalisation (HR = 0.62, 95% CI: 0.42–0.91, *p* = 0.015), with no apparent effect on cardiovascular mortality [18].

The Cochrane systematic review [19], which included 41 randomised controlled trials comparing structured telephone support or non-invasive home telemonitoring to standard care, revealed that both telemedicine modalities significantly reduced the risk of all-cause mortality (by 13% and 20%, respectively) and HF-related hospitalisations (by 15% and 29%, respectively). The positive effects were also expressed in improved health-related quality of life, HF knowledge, and self-care behaviours [19]. Lin et al. [20] extracted data from 39 randomised controlled trials and showed that in HF patients, home-based teletransmission and telephone-supported care reduced all-cause mortality (by 20%), HF-related hospital admissions (by 37%), length of hospital stays, and mortality. The meta-analysis by Zhu et al. [21] presented consistent results, reporting the beneficial effects of telemedicine strategies on all-cause hospitalisation, cardiac hospitalisation, all-cause mortality, and cardiac mortality. The positive effect of telemonitoring was also confirmed by Hafkamp et al. [22], who found a lower risk of HF-related hospitalisations (HR = 0.86, 95% CI: 0.81–0.92, *p* < 0.001). Drews et al. [5] summarised only studies in patients with HF decompensation within the previous month with intervention based on using telemonitoring, defined as the regular transmission of at least one physical variable at least once weekly. The relative risk in the telemonitoring group compared with standard care was neutral for both all-cause hospitalisation (HR = 0.95, *p* = 0.430) and all-cause death (HR = 0.83, *p* = 0.170). However, the authors identified significant clinical heterogeneity among the studies. The qualitative analysis suggested that the effect was in favour of telemonitoring when (1) the telemedicine intervention was relatively simple and easy to use, (2) there was good patient adherence to the intervention, and (3) the therapy was guided by telemonitoring results [5]. The need for solutions that require minimal engagement from users was also emphasised by Berry et al. [23], who found that the majority of the exclusion/inclusion criteria of telemonitoring studies resulted in enrolling patients who were more likely to comply with remote care, as well as overestimation of compliance rates.

## 3. Invasive Remote Monitoring

Almost half of all patients discharged from the hospital are not optimally decongested, and volemic control is crucial to prevent HF rehospitalisation [1,24]. In the CHAMPION-HF study, diuretic therapy based on pulmonary artery pressure monitoring using the CardioMEMS implantable monitor (Abbott Vascular) was evaluated. This approach was revealed to be safe and resulted in a 39% reduction in HF readmissions [25,26]. In the MEMS-HF study, patients’ quality of life and depression were also improved using this therapeutic strategy [27]. In the overall analysis of the recent GUIDE-HF trial [28], conducted in 1000 HF patients with either a recent HF hospitalisation or elevated natriuretic peptides, CardioMEMS-guided HF management failed to reduce the incidence of a composite endpoint (mortality and total HF events) compared to the control group (HR 1.11, *p* = 0.530). However, the pre-coronavirus disease 2019 (COVID-19) impact analysis indicated a possible benefit of this form of telemonitoring on the primary outcome (HR 0.81, *p* = 0.049). This effect was mainly driven by a lower HF hospitalisation rate compared with the control group. According to current guidelines, monitoring pulmonary artery pressure using a wireless haemodynamic monitoring system may be considered in symptomatic HF patients to improve clinical outcomes (recommendation class IIb) [1].

Cardiac-implanted electronic devices (CIEDs) offer another option for invasive remote management of HF patients. The first studies focused on intrathoracic impedance alone, but most failed to provide strong evidence to identify patients at risk of HF deterioration [29,30,31]. However, in the PARTNERS-HF trial, the use of the fluid index combined with other parameters (i.e., atrial fibrillation duration, rapid ventricular rate during atrial fibrillation, low patient activity, abnormal autonomics, and notable device therapy) effectively predicted HF hospitalisation with pulmonary signs or symptoms within the next month (HR = 5.5, *p* < 0.0001) [32]. Additionally, other complex algorithms have proven their clinical utility. Ziele et al. [33] assessed the multisensor remote ambulatory diagnostic risk score TriageHF™ in a real-world, unselected, large patient sample (*n* = 22,901), and they concluded that this tool correctly classified the patients into risk categories according to HF events and all-cause mortality. Another multiparameter tool—the HeartLogic algorithm—allowed relevant HF-related clinical conditions to be identified remotely, with low rates of unexplained alerts and undetected HF events [34].

In the IN-TIME study [35], automatic daily multiparameter telemonitoring of CIEDs resulted in a better prognosis for patients with chronic HF. The difference in the composite primary outcome (i.e., all-cause death, overnight hospital admission for HF, change in New York Heart Association class, and change in patient global self-assessment) between the telemonitoring group and standard care was clinically relevant (18.9% versus 27.2%, *p* = 0.013). In the EFFECT trial, remote monitoring of CIEDs resulted in a significant reduction in the primary endpoint (i.e., the rate of death and cardiovascular hospitalisations, incident rate ratio (IRR) = 0.55, *p <* 0.001) [36]. The 2016 European Society of Cardiology (ESC) Guidelines for Heart Failure Diagnosis and Treatment [37] stated that multiparameter monitoring of CIEDs may be considered in symptomatic patients with HF (LVEF < 35%) to improve clinical outcomes (recommendation class IIb). However, the authors of the 2021 edition [1] are more conservative; they admit strong evidence for remote monitoring to detect device malfunction arrhythmias, but they concur that there is little evidence that device monitoring reduces HF admissions or mortality. However, in a recently published state-of-the-art review of implanted haemodynamic monitoring devices for patients with heart failure, including a meta-analysis of RCTs, Iaconelli et al. [38] revealed that haemodynamic monitoring reduced hospitalisations for HF (HR = 0.75; 95% CI 0.58–0.96; *p* = 0.030), but not mortality (*p* = 0.480).

## 4. Non-Invasive Monitoring of Volume Status

There have also been attempts to improve the prognosis of HF patients by using remote non-invasive haemodynamic assessment of volemia. It has been reported that when measured non-invasively, lung impedance (LI) enables the detection of preceding HF exacerbations 2–4 weeks before symptoms occur [39]. This technique was developed to calculate the net lung impedance, and the assessment of congestion is based on the assumption that air and water have different resistance to applied current. Three electrodes are placed vertically on the front upper-right side of the chest, and another three electrodes are placed on the back along the horizontal line, below the right scapula [39]. However, it should be emphasised that the applied electrode topography limits the assessment of lower lung segments, where fluid accumulation is most likely to occur first.

In the IMPEDANCE-HF trial [40] involving patients after an episode of acute HF, LI-guided treatment was associated with a lower HF hospitalisation rate (HR = 0.63, *p* < 0.001), as well as a reduction in total deaths (HR = 0.52, *p* = 0.002), cardiovascular deaths (HR = 0.41, *p* < 0.001), and deaths due to HF (HR = 0.35, *p* = 0.001). The secondary analysis showed that LI-based assessment of pulmonary fluid predicted 30- and 90-day HF readmissions better than other variables, such as N-terminal pro-brain natriuretic peptide, weight, radiological score, New York Heart Association class, lung rales, leg oedema, or jugular venous pressure (*p* < 0.01) [41]. Another system for assessing lung hydration stems from electromagnetic-energy-based technology [42]. This method is based on the measurement of the dielectric properties of tissues exposed to a low-energy electromagnetic field, and the assumption that it will be sensitive to changes in the water content in lung tissue. In a prospective, randomised, and controlled study, the ReDS system (SMILE™)-guided therapy, when compared to usual care, resulted in a 48% reduction in readmissions (HR = 0.52, *p* = 0.010) and fewer days lost to acute decompensated HF hospitalisation (1.37 vs. 2.62 days, *p* = 0.006), but no significant influence on mortality [43]. In another study, patients identified as having residual congestion at discharge (ReDS ≥ 39%) had a higher 30-day readmission rate compared to those who were adequately decongested (11.8% vs. 1.4%, *p* = 0.030). However, the ReDS-guided intensified diuretic therapy did not prevent HF readmission [44].

There are also encouraging reports on the use of bioimpedance spectroscopy, showing the usefulness of this method in the detection of congestion and the early prediction of HF deterioration [45,46].

ESC experts present a conservative attitude towards this evidence and state that there is still uncertainty as to whether these remote technologies for lung congestion offer additional benefits over conventional home telemonitoring [1].

## 5. Virtual Wards

Telemedicine solutions have also been incorporated into the idea of virtual wards (VWs) for patients at high risk of readmission or death after discharge. This model of care is a combination of telephone, home, and/or clinic visits. Dhalla et al. [47] compared this therapeutic approach to standard care and did not observe a statistically significant difference for the composite endpoint of hospital readmission or death within 30 days of discharge (21.2% vs. 24.6%, *p* = 0.090). Low et al. [48] integrated the outpatient VW team (physician and nurse) with an inpatient care team (physician, medical officer, nurse and pharmacist) working together on the same electronic patient record. This modified model, compared to standard care, resulted in a significant reduction in the 30-day readmission rate (IRR = 0.67, *p* = 0.001) and in the number of emergency department visits at 30 days (IRR = 0.60, *p* < 0.001). The meta-analysis by Uminski et al. [49] revealed that in patients with HF, post-discharge VWs as an alternative to usual community-based care reduced the risk of mortality (risk ratio (RR) = 0.59, *p* < 0.001) and HF readmissions (RR = 0.61, *p* < 0.001). Friedman et al. [50] described the effects of virtual cardiology consultations in the COVID-19 era (between August 2020 and February 2021) on the outcomes of 3510 HF patients discharged to skilled nursing facilities. This retrospective analysis revealed lower hospital readmission among patients who received one or more virtual consultations compared with the expected readmission rate for both cardiac (3% vs. 10%, respectively) and all-cause aetiologies (18% vs. 27%, respectively). The investigation provided initial evidence for the potential effectiveness and efficiency of digitally enabled virtual cardiovascular care on 30-day hospital readmissions. A recently published meta-analysis of 11 clinical trials in patients with HF revealed that VWs were associated with lower mortality (RR = 0.86; 95% CI 0.76–0.97, *p* = 0.020) and fewer HF readmissions (RR = 0.84; 95% CI 0.74–0.96, *p* = 0.008) [51].

## 6. Telerehabilitation

Exercise training is recommended for HF patients to improve their exercise capacity and quality of life, and to reduce HF hospitalisations [1]. Cardiac rehabilitation programmes should be especially considered in high-risk patients and implemented after hospitalisation for acute cardiac events [52,53]. Advancements in telemedicine have provided the potential to create safe, effective, and standardised home-based cardiac telerehabilitation programmes [53,54]. In a multicentre, prospective, parallel-group randomised TELEREH-HF trial that enrolled 850 patients with HF up to 6 months after a cardiovascular hospitalisation, Piotrowicz et al. [55] demonstrated positive effects of a 9-week programme of hybrid comprehensive telerehabilitation on peak oxygen consumption and quality of life in patients with HF. However, no improvements in the percentage of days alive and out of the hospital, mortality, and hospitalisation were noted in the long-term (14–26 months) follow-up period [55]. Cavalheiro et al. [56] conducted a systematic review and meta-analysis of randomised controlled trials (overall 2206 patients) and showed cardiac telerehabilitation to be effective in improving HF patients’ functional capacity (as measured using the 6-minute walk test and peak oxygen uptake) and quality of life. No major adverse events were reported during the remotely monitored exercise. However, only 1 of 17 studies presented data on mortality during the follow-up, and only 4 reported HF hospitalisations. Insernia et al. [54] included 19 RCTs in their review and revealed a reduction in the risk of HF hospitalisation by 23%, but without any effect on mortality.

In view of the known limitations of conventional cardiac rehabilitation (e.g., lack of resources, as well as logistical and psychological problems), telerehabilitation could be the next step in providing this beneficial component of care to more HF patients.

## 7. Prognostic Digital Algorithms

Effective post-discharge planning is an important component of care in HF [1]. There have been attempts to support this process with digital algorithms, including AI—especially with regard to risk stratification. Allain et al. [57] designed an electronic personalised discharge checklist to screen elderly HF patients for comorbidities and provide adequate therapy. The authors noted a non-significant trend towards a reduction in the primary composite outcome of mortality and HF readmissions (HR 0.79, *p* = 0.150). Moreover, the management of HF comorbidities was significantly improved. Fahimi et al. [58] developed a recurrent neural network model for dynamic risk prediction based on data acquired within a non-randomised controlled study that enrolled 150 HF patients over a 1-year post-discharge telemonitoring and telesupport programme. The model detected emerging readmissions with a sensitivity > 71% and specificity > 75%. Romero-Brufau et al. [6] tested AI-based predictive modelling with a clinical decision-support intervention to reduce unplanned hospital HF readmissions in a non-randomised prospective trial. Each patient’s individual report identified a risk category, risk factors, and targeted recommendations intended to address the identified risk factors. There were 26 possible recommendations that could be generated by the tool. In the 6 months following implementation, readmission rates decreased from 11.4% during the comparison period to 8.1% (*p* < 0.001). After accounting for the 0.5% decrease in readmission rates (from 9.3 to 8.8%) at control hospitals, the relative reduction in the readmission rate was 25% (*p* < 0.001). In the LINK-HF multicentre study [59], a personalised analytical platform using continuous data streams to predict HF exacerbations was evaluated. The measurements from a wearable patch (i.e., electrocardiogram, heart rate, respiratory rate, body temperature, body position, and activity levels) were uploaded continuously via smartphone to a cloud-based machine learning algorithm. The platform predicted rehospitalisations with 76–88% sensitivity and 85% specificity, with a median time of 6.5 days between the initial alert and readmission. A prospective randomised study to determine whether therapy based on this approach can reduce HF rehospitalisation rates is ongoing (NCT04502563). Croon et al. [60] analysed 23 articles on AI-based algorithms for the prediction of hospital admissions in patients with HF, and they reported its encouraging performance in the prediction of HF readmissions in short- and long-term follow-up (area under the curve for 30 days ranging from 0.61 to 0.79, and for 6 months–3 years ranging from 0.65 to 0.78). The use of telemonitoring data from disposable sensory patches strongly improved the algorithms’ performance.

## 8. Patients’ and Healthcare Providers’ Perspectives

The COVID-19 pandemic has accelerated the adoption and acceptance of telemedicine technologies in cardiovascular disease management worldwide [61,62,63]. Generally, virtual consultations are accepted and practical compared to in-person visits. However, it is reported that older patients prefer face-to-face appointments [64]. Patients are aware that telehealth may provide benefits regarding such perspectives of care as location, access, and efficiency [65]. Telemedicine is perceived as convenient and time-saving due to reducing the time spent travelling and waiting [66].

The future role of specialised websites and database repositories that store patients’ data may be crucial for the continuity of care, especially during pandemics and times of limited access to healthcare resources. This idea is strongly supported by medical professionals [67], as well as by patients, who in some countries would even accept paying for telemedicine pharmaceutical care services [68]. Digital health technologies may support healthcare through several useful tools, such as teleconsultations, e-diagnosis, e-prescription, and e-referral [69]. E-prescription is already used in many countries, improving the standard of patient care. Most patients recognise that e-prescription makes it easier to purchase medications and are positive about obtaining prescriptions for continued treatment without a personal doctor visit [70].

However, there are several concerns regarding the use of remote solutions from the patient’s perspective, including privacy, data protection, challenges with technology (especially internet connectivity, technology literacy, etc.), and the lack of physical contact (decreasing nonverbal communication and other humanistic aspects). To ensure a positive patient experience, one should assess their capacity to use the proposed technology and provide detailed information and instructions. The ease of contacting the clinic and scheduling the in-person appointment should be also ensured [64].

Successful implementation of telemedicine requires multisectoral engagement of stakeholders (e.g., government, civil society, healthcare providers). Appropriate strategies, especially with regard to nationwide deployment and stable funding, are inducements to fast adaptation.

Undoubtedly, the evidence of clinical effectiveness will be the most important incentive for the use of telemedicine technologies. However, to ensure healthcare workers’ acceptance of remote solutions, some important issues should be also considered, including users’ culture, education, and motivation; the diagnostic accuracy of the technology; user technology literacy and satisfaction; infrastructure; easily accessible technical support; cost-effectiveness; and additional (or decreased) work burden [71].

## 9. Recommendations, Knowledge Gaps, and Challenges

Although it has been proposed that some telemedicine modalities can be implemented to ensure continuity of care for patients early after an event of acute HF, the current guidelines are still conservative. This is mainly because of scarce evidence from multicentre prospective trials. According to the ESC (European Society of Cardiology) guidelines for the diagnosis and treatment of HF, non-invasive home telemonitoring may be considered for patients with HF to reduce their risk of cardiovascular and HF hospitalisations and cardiovascular death (class IIb). Invasive telemonitoring through pulmonary artery pressure sensors is recommended to be considered in symptomatic patients to improve clinical outcomes (class IIb) [1]. Telephone support and telemonitoring are also mentioned as important elements of HF management programmes for the follow-up of patients after discharge. The experts also perceive home telemonitoring as an effective method for providing patient education and motivation. The adaptation of existing healthcare systems is definitely recommended [1]. The AHA/ACC/HFSA (American College of Cardiology/American Heart Association Science/Heart Failure Society of America) guidelines consider wireless monitoring of pulmonary artery pressure in selected symptomatic patients with NYHA (New York Heart Association) as class III, with maximally tolerated pharmacotherapy and optimal device therapy (class IIb), with the comment that the value of this method for patient prognosis is still uncertain. The experts conclude that further studies on these approaches are definitely needed before they can be recommended for routine clinical care [72].

**Figure 1:**
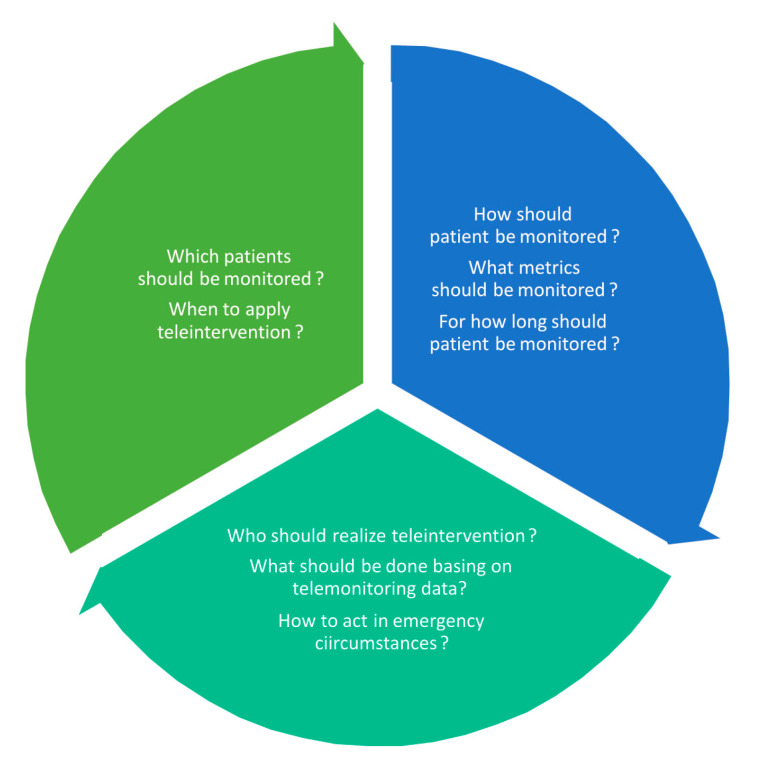
Dilemmas concerning the use of telemedicine solutions.

Future research should address still-existing dilemmas regarding the use of telemedicine solutions in HF patients who have recently been discharged from hospitals (Figure 1). The proper selection of candidates is important not only from a clinical perspective, but also in view of optimal resource management and costs. Not every patient would benefit from telemedicine—not only because of their variable technology literacy, but also due to some clinical features that may limit the potential benefits (e.g., advanced stage of disease, depression, poor prognosis, non-compliance). The time of teleintervention should be also adapted to the course of the disease. The remote support applied in the vulnerable phase does not have to be obligatorily extended to a lifelong process in all patients. The set of telemedicine modalities should also be individually selected. Not all patients need a full set of available telemonitoring devices. Similarly, the intensity and timeframe of remote control could be flexibly scheduled. The increasing application of algorithms, machine learning techniques, and artificial intelligence is also expected soon. The management of acquired data is crucial and, at the same time, challenging. The organisation of telemedicine centres demands precise assignment of personnel tasks, standard operating procedures, and clear instructions of how to act in emergency circumstances.

## 10. Conclusions

The COVID-19 pandemic has accelerated the adoption and acceptance of telemedicine technologies in cardiovascular disease management. There are robust proposals that some digital solutions could be implemented at present to ensure continuity of care for patients early after events of acute HF. Further rapid progress in this area is expected. However, due to existing research gaps and challenges, future randomised telemedicine trials and real-life registries are still definitely needed.

## Figures and Tables

**Table 1:** Digital health technologies used in heart failure.

Telecare and remote disease management
Video (tele) consultations, virtual visits, virtual wards; Structured telephone support, automated voice response systems; Transmission of information on symptoms and signs (e.g., apps); Cardiac telerehabilitation; Tele-education, remote psychological support.
Telemonitoring
Invasive remote monitoring (e.g., CIEDs, haemodynamic implanted monitors); Non-invasive remote monitoring of vital signs —standalone and wearable devices (e.g., blood pressure, heart rate, weight, oxygen saturation, electrocardiogram, volemia, glucose, activity).
Supporting digital tools
Electronic medical records; Electronic telemedicine platforms, cloud-based platforms; Computer algorithms, machine learning, artificial intelligence (risk stratification, diagnosis, recommendation support).

## Data Availability

Not applicable.
